# Case of Diphtheria Treated by Tracheotomy, Peptonised Enemata and Iodoform

**Published:** 1884-03

**Authors:** R. Shingleton Smith

**Affiliations:** Physician to the Bristol Royal Infirmary


### CASE OF DIPHTHERIA TREATED BY TRACHEOTOMY, PEPTONISED ENEMATA AND IODOFORM. By R. SHINGLETON SMITH, M.D., M.R.C.P., Physician to the Bristol Royal Infirmary.

THE following case of recovery after Tracheotomy for Laryngeal Diphtheria is worthy of short record.

Florence H., æt. 6, admitted Dec. 26th, 1883, in state of asphyxia, with abundant exudation visible in throat. Tracheotomy was performed immediately, and gave marked and immediate relief.

Dec. 27.—Temperature was 102°, urine had one-eighth albumen, and there was much swelling of cervical glands.

Dec. 30.—The edges of the wound were gaping, stitches had sloughed out, and glands very much swollen.

Dec. 31.—Milk taken in by mouth at once came out through the tracheal tube and gave rise to much choking cough. Nutrient enemata of peptonised milk, and Slinger's capsules were given; all food by the mouth discontinued.

Jany. 4.—The child was wasting rapidly, although the temperature was normal, and threatened pneumonia had cleared up. The enemata not being well retained peptonised milk was injected into the stomach through an œsophageal tube, but was only partially retained. The tube in trachea was then removed; the sloughing of wound had ceased, the swelling of glands subsided. In a few days the child could swallow solid food, although liquids would still pass down the larynx. The wound in the neck gradually healed, the opening in trachea closed, power of deglutition both of solids and liquids returned. The child rapidly improved in strength; speech returned in about a month, and the urine was free from albumen six weeks after admission.

The only essential element of treatment, other than by tracheotomy, peptonised enemata and œsophageal injections, was iodoform. This was given in grain doses with mucilage every four hours during the first five days, till the power to swallow was lost. Iodoform was also painted on the fauces, and diffused in vapour throughout the air of the steam-bed. It was also applied to the wound and over the tube in the form of iodoform wool.

